# Insights from the supplementary motor area syndrome in balancing movement initiation and inhibition

**DOI:** 10.3389/fnhum.2014.00960

**Published:** 2014-11-28

**Authors:** A. R. E. Potgieser, B. M. de Jong, M. Wagemakers, E. W. Hoving, R. J. M. Groen

**Affiliations:** ^1^Department of Neurosurgery, University Medical Center Groningen, University of GroningenGroningen, Netherlands; ^2^Department of Neurology, University Medical Center Groningen, University of GroningenGroningen, Netherlands

**Keywords:** supplementary motor area (SMA), supplementary motor area syndrome, akinetic mutism, neurosurgery, Parkinson’s disease, tic disorders

## Abstract

The supplementary motor area (SMA) syndrome is a characteristic neurosurgical syndrome that can occur after unilateral resection of the SMA. Clinical symptoms may vary from none to a global akinesia, predominantly on the contralateral side, with preserved muscle strength and mutism. A remarkable feature is that these symptoms completely resolve within weeks to months, leaving only a disturbance in alternating bimanual movements. In this review we give an overview of the old and new insights from the SMA syndrome and extrapolate these findings to seemingly unrelated diseases and symptoms such as Parkinson’s disease (PD) and tics. Furthermore, we integrate findings from lesion, stimulation and functional imaging studies to provide insight in the motor function of the SMA.

## Introduction

The supplementary motor area (SMA) syndrome is a characteristic neurosurgical syndrome that may occur after unilateral resection of the SMA. The classical SMA syndrome, following unilateral resection of the SMA, is characterized by a global akinesia with normo- or hyporeflexia and a normal tonus, more profound on the contralesional side, while muscle strength can be preserved (Laplane et al., 1977). A remarkable feature is that the symptoms completely resolve within weeks to months, only leaving a disturbance in alternating bimanual movements as the remaining deficit (Laplane et al., 1977).

The SMA and its function have been the subject of intensive study (see Nachev et al. (2008)). Here we specifically focus on the lessons learned from the clinically observed SMA syndrome, particularly the motor components. The origin of reflex abnormalities in the SMA syndrome has been described previously (Florman et al., 2013). This review aims to integrate previous findings from lesion and stimulation studies in both monkeys and man with current lesion and neuroimaging studies in patients with an infarct or resection of the SMA.

The SMA or SMA proper (Brodmann area 6) is localized in the posterior part of the superior frontal gyrus (Penfield and Welch, 1951). The cingulate sulcus and gyrus demarcate its inferior border. The posterior SMA border is constituted by the precentral sulcus separating it from the leg area of the primary motor cortex. The lateral and anterior borders are less clearly demarcated on macro-anatomical criteria, although histochemical and cytoarchitectonic differences have been well described (Matelli et al., 1985, 1991; Geyer et al., 1998). Functionally, the position of the SMA has been extensively characterized in a meta-analysis of 126 functional studies (Mayka et al., 2006). Anteriorly the SMA can be distinguished from the pre-SMA, roughly by using the vertical traversing the anterior commissure as a border (Picard and Strick, 2001). The lateral borders are constituted by the dorsal premotor cortex in each hemisphere (Mayka et al., 2006).

The SMA has a somatotopical organization, first described in monkeys (Mitz and Wise, 1987; Luppino et al., 1991), and later confirmed in humans (Fried et al., 1991; Lim et al., 1994; Mayer et al., 2001; Fontaine et al., 2002; Chainay et al., 2004). It has been shown that the face, upper limbs and lower limbs are represented in an anteroposterior direction in the SMA. In the dominant hemisphere, language seems to be represented most anteriorly (Fontaine et al., 2002).

**Figure 1:**
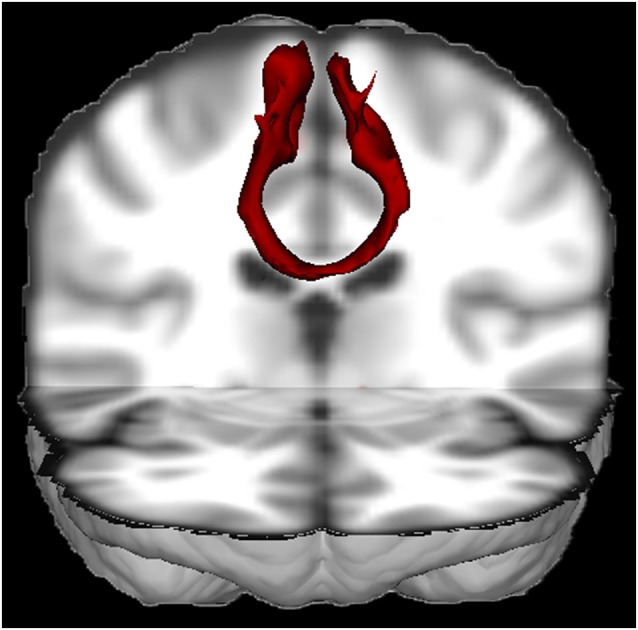
**3D view of the probabilistic tractography between both SMA’s from a single healthy subject (made with FSL)**. The tractography result was transformed to Montreal Neurological Institute (MNI) space. This figure nicely illustrates that the SMA’s are densely interconnected through the corpus callosum.

The SMA is an eloquent area with rich connections to both cortical and subcortical structures. About ten percent of the input from the corticospinal tracts originates in the SMA (Murray and Coulter, 1981; Mitz and Wise, 1987; Dum and Strick, 1991; He et al., 1995; Maier et al., 2002). Furthermore, the SMA is strongly embedded in motor circuits through its connections with the primary motor cortex, premotor cortex and cingulate cortex (Luppino et al., 1993). There are connections with the superior parietal lobe, insula (Luppino et al., 1993), basal ganglia (Inase et al., 1999; Lehéricy et al., 2004; Akkal et al., 2007), thalamus (Behrens et al., 2003), cerebellum (Akkal et al., 2007) and especially with the contralateral SMA (see Figure 1) through the corpus callosum (Liu et al., 2002). Recently, connectivity of the SMA has also been characterized with diffusion tensor imaging (DTI) with post-mortem dissection as a validation method (Vergani et al., 2014). Vergani et al. (2014) confirmed the recent notion that the SMA is also connected with the pars opercularis of the inferior frontal gyrus (Broca’s area) through the frontal aslant tract (Ford et al., 2010; Catani et al., 2012).

**Figure 2:**
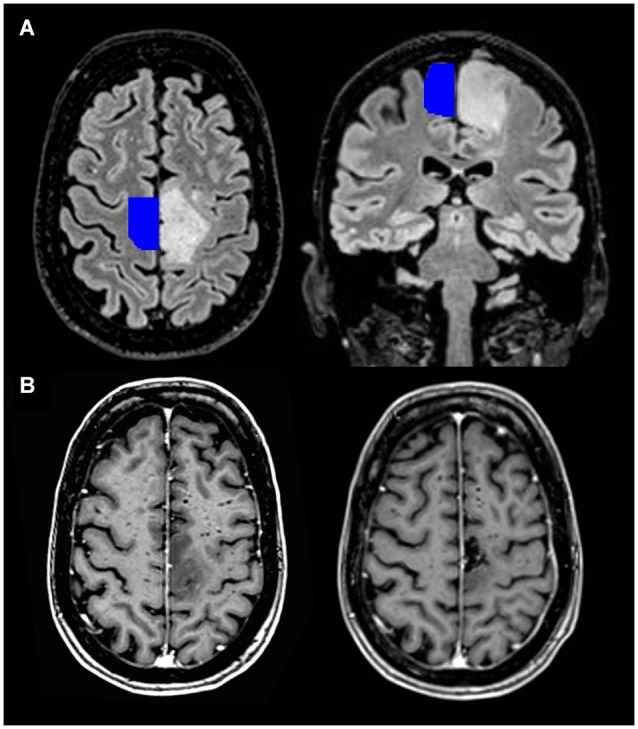
**3D view of the probabilistic tractography between both SMA’s from a single healthy subject (made with FSL).**^1^ Pre- and postoperative MRI scan of a 64-year-old patient with a diffuse astrocytoma (WHO grade II) in the left SMA. **(A)** Transversal and coronal T2-weighted FLAIR images, with an SMA template projected on the healthy hemisphere. The latter is freely available and derived from a large meta-analysis describing the location of the sensorimotor areas (Mayka et al., 2006). **(B)** Transversal images after gadolinium contrast from the same patient before (left lower corner) and three months after the operation (right lower corner). She had a complete motor loss on the right side after the operation, which quickly recovered.

Since its initial recognition, the SMA syndrome has remained elusive. Although described earlier, Laplane et al. (1977) were the first to link the symptoms to a syndrome caused specifically by SMA removal (Laplane et al., 1977). By definition, sensory functions remain undisturbed. Reduced spontaneous speech may occur, predominantly described after resection of the SMA in the dominant hemisphere, though not exclusively (Laplane et al., 1977; Bleasel et al., 1996; Ulu et al., 2008). Most often, strict lesions of the SMA do not result in a specific class of aphasia. Cases have been described of patients with unilateral resections of the SMA or an ischemic lesion that presented with mutism or reduced speech without signs of aphasia (Bleasel et al., 1996; Krainik et al., 2003; Mendez, 2004). Transcortical motor aphasias have been described, but it is more plausible that these are the result of more extensive damage to the subcortical white matter (Freedman et al., 1984). Possibly, the frontal aslant tract is affected, which is more lateralized to the left hemisphere (Vergani et al., 2014), explaining the more frequent occurrence of additional linguistic deficits after left SMA resections. Although it remains uncertain whether more anterior SMA resections can result in a specific aphasia, we will not further focus on that. In the weeks to months following resection of the SMA there is reduced movement and speech. This syndrome almost always completely resolves, although minor deficits in alternating movements of upper and lower limbs have been observed to remain (Laplane et al., 1977). Although a further parcellation into SMA and pre-SMA was proposed later (Tanji, 1994), many cases of patients with a consistent symptomatology complex have been described (Rostomily et al., 1991; Bleasel et al., 1996; Zentner et al., 1996; Bannur and Rajshekhar, 2000; Duffau et al., 2001; Krainik et al., 2001, 2003, 2004; Fontaine et al., 2002; Nelson et al., 2002; Peraud et al., 2002; Russell and Kelly, 2003; Yamane et al., 2004; Ulu et al., 2008; Rosenberg et al., 2010; Martino et al., 2011; Tate et al., 2011; Kasasbeh et al., 2012; von Lehe et al., 2012; Kim et al., 2013; Schucht et al., 2013), showing that 11–100% of the patients develop the SMA syndrome after unilateral resection of the SMA. Similar cases have been described, for example, in the context of infarction (Pai, 1999; Kumral et al., 2002; Radman et al., 2013) or embolization of an arteriovenous malformation (Schell et al., 1986), having SMA involvement in common. In neurosurgical practice, presentation of the SMA syndrome after SMA removal may cause major concerns, due to the fear of possible corticospinal tract damage. Although symptoms can be mild and are transient, this syndrome is a significant burden in brain tumor patients that already have a shortened life expectancy. Figure 2 shows a preoperative MRI scan of a patient that developed the SMA syndrome after resection of a tumor, with a template of the localization of the SMA projected on the healthy hemisphere (Mayka et al., 2006).

In this article, we focus on the motor aspects of the SMA syndrome and what can be learned about the motor function of the SMA from this intriguing syndrome. We extrapolate these findings to seemingly unrelated diseases and symptoms such as Parkinson’s disease (PD) and tics. Combining these findings, we propose that the SMA is involved in both the initiation and suppression of movements, maintaining a tonic interhemispheric balance.

## Cause of the SMA syndrome

A hallmark of the SMA syndrome that is always described is a severe neurological deficit of temporary nature; only subtle deficits are permanent. Although the precise mechanisms underlying the recovery after the initial deficit remain obscure, the syndrome provides useful insights in the functioning of the SMA. The occurrence of the different deficits of the SMA syndrome following resection is consistent with the somatotopical organization of the SMA (Fontaine et al., 2002; Krainik et al., 2004) and the deficits are correlated with the extent of resection of functionally active SMA (Krainik et al., 2001, 2003, 2004). There is an association between neurological deficit and the distance from the resected area to the SMA (Nelson et al., 2002), to the precentral sulcus (Peraud et al., 2002; Kasasbeh et al., 2012) and the cingulate sulcus (Kasasbeh et al., 2012; Kim et al., 2013). Also, an increased incidence of the SMA syndrome and the severity of symptoms is seen when the anteroposterior extent of resection is larger (Zentner et al., 1996; Krainik et al., 2001; Ulu et al., 2008; Kasasbeh et al., 2012). Russell and Kelly (2003) showed that both a resection larger than 90% and the presence of a low-grade glioma are associated with a higher incidence of the SMA syndrome (Russell and Kelly, 2003). They argued that residual function of the SMA is still present in patients harboring a low-grade glioma, while it is unlikely that the SMA syndrome develops in patients with high-grade gliomas, due to the absence of functional neural tissue inside these tumors (Russell and Kelly, 2003). A very intriguing finding was observed in a patient undergoing awake surgery during which the SMA syndrome occurred with a delay of half an hour after the resection (Duffau et al., 2001). The authors suggested that an initial compensation of function is possible due to parallel networks or due to residual activity of an oscillatory loop that supports the execution of function but not its initiation (Duffau et al., 2001). The case of Duffau et al. (2001) provided new evidence about underlying mechanisms (Duffau et al., 2001). They made clear that it is highly unlikely that this syndrome is caused by venous thrombosis or postoperative edema, because symptoms presented too early for that (Duffau et al., 2001). A follow-up MRI showed no signs of ischemia or venous thrombosis. Edema is also unlikely because it takes weeks to months for the deficits to restore. Furthermore, as noted before, the SMA syndrome has also been described to result from other disease mechanisms such as following an infarct.

## Mechanisms of recovery

Effort has been undertaken to understand the mechanisms underlying the recovery. Functional reorganization due to brain plasticity has been brought up in order to understand the temporary deficits. A lesion in the SMA leads to more activation of the contralateral SMA (Sailor et al., 2003). However, it is uncertain whether this reflects functional compensation or is merely the consequence of decreased transcallosal inhibition from the damaged hemisphere (Shimizu et al., 2002). In patients with left dominant hemisphere lesions in language areas high-frequency repetitive transcranial magnetic stimulation (rTMS) over the right hemisphere disturbs language function in patients with left dominant hemisphere lesions in language areas, which shows that activation in the contralateral hemisphere truly represents function (Thiel et al., 2005), rather than mere loss of transcallosal inhibition. Others have shown that a preoperative switch in activation to the contralateral healthy SMA is not sufficient to avoid the syndrome (Rosenberg et al., 2010), but leads to a faster recovery (Krainik et al., 2004). This is supported by the fact that the SMA has strong connections with its contralateral counterpart (Rouiller et al., 1994; see also Figure 1). Others have raised that hemispheric dominance of the SMA might be important in predicting postoperative deficits (Nelson et al., 2002), which could explain why not everyone develops the SMA syndrome after unilateral resection of the SMA. However, there is no substantial evidence that provides convincing support for this argument. A relation between the side of the resection and incidence of the syndrome has not been described. Postoperatively, the functional recruitment of the healthy SMA and premotor cortex seems to compensate for the resection of the SMA (Krainik et al., 2004).

In summary, clinical deficits after resection of the SMA may vary from none to a global akinesia with mutism. On the one hand, this finding emphasizes the heterogeneity associated with lesion studies, particularly in cerebral infarcts, but also after resection of tumors that are not always completely restricted to the SMA. On the other hand, the heterogeneity in clinical symptoms after resections may be caused by variability in preoperative reorganization of function due to brain plasticity.

It is evident that preoperative reorganization of cerebral function does not completely account for the recovery, because the reversibility of the SMA syndrome is also seen in patients with acute lesions such as an infarct or patients that undergo surgery for epilepsy (Yamane et al., 2004; Kasasbeh et al., 2012). It is plausible that the patient population with slow growing lesions and subsequent acute surgical lesion have a tendency to recover faster due to preoperative reorganization (Desmurget et al., 2007), although this is yet to be proven for the SMA syndrome. Another possibility is an additional functional distortion of the SMA due to the mass effect of tumors. A resection alleviates this compression, which uncovers residual function of the affected SMA (if any).

## Bimanual movement patterns

It is remarkable that although the other more striking deficits of the SMA syndrome completely resolve, difficulties in alternating bimanual movements persist. We focus on bimanual alternating movements here, because this impairment is well described in patients with the SMA syndrome. Disturbed alternating movements of the lower limbs have also been described (Laplane et al., 1977), but most of the time lower limb function is not documented. Although SMA lesions in monkeys do not result in the typical SMA syndrome as seen in humans (Travis, 1955), these primates do have deficits in bimanual coordination (Brinkman, 1981). The bimanual coordination deficit after unilateral lesioning of the SMA (and most likely also including the pre-SMA at that time) was resolved after callosal sectioning, suggesting that the intact SMA influenced the motor program for both hands (Brinkman, 1984). Brinkman (1984) even described a monkey that behaved as having two preferred hands after resection of the non-dominant SMA and subsequent callosal section (Brinkman, 1984). A persistent disturbance in bimanual alternating movements has also consistently been described in patients with the SMA syndrome (Laplane et al., 1977), during which the hand contralateral to the lesion is the one that seems to be at fault (Bleasel et al., 1996). This is possibly the result of the fact that alternating bimanual movements are cognitively more demanding than mirror movements. There is a preference for simultaneous rather than alternating bimanual movements with increasing frequency of movements (Kelso, 1984; Lee et al., 1996). Such simultaneous movements are more stable and performed more accurately (Yamanishi et al., 1980; Swinnen et al., 1997; Stephan et al., 1999a; Meyer-Lindenberg et al., 2002). 5 Hz rTMS of the SMA causes a disturbance in both in- and anti-phase movements, although the latter is more evidently disturbed (Serrien et al., 2002). There is ample evidence of enhanced SMA activation during anti-phase movements (Sadato et al., 1997; Goerres et al., 1998; Stephan et al., 1999b; Toyokura et al., 1999; Immisch et al., 2001; Ehrsson et al., 2002; Meyer-Lindenberg et al., 2002; Ullén et al., 2003; Debaere et al., 2004; Kraft et al., 2007; Goble et al., 2010; Wu et al., 2010) and this does not seem to be restricted to the bimanual character of anti-phase movements (Koeneke et al., 2004). The SMA is definitely neither the sole contributor nor specific for bimanual coordination (Kazennikov et al., 1999; de Jong et al., 2002; Aramaki et al., 2006; Grefkes et al., 2008; see also Swinnen (2002) for a review), but the SMA syndrome provides evidence that bilateral functioning of the SMA is a requirement for anti-phase movements. Only for anti-phase movements there is a difficult balance between initiation of the motor task and contralateral suppression (Stephan et al., 1999a). A bilateral contribution of the SMA to bimanual coordination has also been shown by direct stimulation during surgery (Martino et al., 2011). It has been hypothesized that the opposite SMA rapidly takes over the motor function for both sides of the body (Martino et al., 2011). However, for the execution of bimanual alternating movements function of both SMA’s is necessary. In the last paragraph we will return to the issue why particularly a disturbance in alternating bimanual movements persists in the SMA syndrome.

## Comparison with impaired/altered SMA function in Parkinson’s disease and tics

Although PD is a chronic deteriorating disease and the SMA syndrome is acute, some parallels can be seen between these disorders. PD is caused by a loss of dopaminergic neurons in the pars compacta of the substantia nigra (Gibb and Lees, 1991). At the cortical level, decreased activity of the SMA has been well recognized (Playford et al., 1992; Eidelberg et al., 1994; Grafton, 2004), which can be improved with deep brain stimulation of the subthalamic nucleus (Grafton et al., 2006) or treatment with levodopa (Haslinger et al., 2001; Buhmann et al., 2003). Similarly, treatment with apomorphine causes an improvement in the impaired activation of the SMA (Jenkins et al., 1992). Thus, the reduced output from the basal ganglia in PD most likely leads to a functionally impaired SMA that can be improved with conventional treatment methods. This is consistent with the observed decrease in the “Bereitshaftspotential” that occurs in PD, further supporting the concept that disturbed SMA functioning leads to a deficit in voluntary movements (Nachev et al., 2008). The Bereitshafspotential has been shown to increase prior to sequential movements (Benecke et al., 1985).

As in the SMA syndrome, patients with PD show a disturbance in the performance of alternating movements (Dick et al., 1986; Benecke et al., 1987; Jones et al., 1992). Moreover, patients with PD can perform normal in-phase movements, while they are specifically less proficient in bimanual anti-phase movements (Johnson et al., 1998; Serrien et al., 2000; Geuze, 2001; Almeida et al., 2002; Ponsen et al., 2006; Wu et al., 2010), which is accompanied by decreased SMA and basal ganglia activation compared to healthy controls (Wu et al., 2010). Both disorders can be characterized by akinesia (Jankovic, 2008). Patients with the SMA syndrome are able to perform normal movements when strongly encouraged to do so (Laplane et al., 1977). This very interesting finding suggests that a different circuit may take over the role of the SMA. Such circuitry might similarly be expected to compensate for the disturbed functioning of the SMA in patients with PD. Bilateral extirpation of the SMA in monkeys leads to akinesia, without deficits in movement time, reaction time, or motivation (Passingham, 1993). However, subsequent experiments showed that the monkeys are impaired in the execution of appropriate movements only in the absence of external cues (Passingham, 1993). The monkeys are able to restore from this deficit, for which the lateral premotor cortex is possibly accountable (Passingham, 1993).

The SMA has been shown to be active during the selection of movements and word generation when there are no external cues, while the lateral premotor cortex is activated when there are cues (Passingham, 1993; Crosson et al., 2001). On the other hand, neurons in the lateral premotor cortex can also respond to self-initiated tasks without external cues (Romo and Schultz, 1987; Kurata and Wise, 1988). For patients with PD, akinetic starting difficulties can be resolved with external cues (kinesia paradoxa; Jankovic, 2008). Furthermore, micrographia in patients with PD can be temporarily improved upon encouragement (McLennan et al., 1972; Oliveira et al., 1997). Equivalent to the SMA syndrome, PD patients do not seem to have dysfunction of the lateral premotor cortex (Playford et al., 1992; Jahanshahi et al., 1995). Patients with PD showed relatively decreased SMA activity during a sequential finger movement task, while there was increased activity in the lateral premotor cortex in both hemispheres (Samuel et al., 1997). Analogously, as mentioned in a previous paragraph, recruitment of the lateral premotor cortex was seen in the healthy hemisphere in patients after unilateral resection of the SMA. Such recruitment increased with the extent of tumor infiltration in the SMA (Krainik et al., 2004).

Indeed, the pathophysiology underlying the SMA syndrome and PD are completely different. Nevertheless, the phenomenology can help in understanding the function of the SMA. For example, a patient has been described with a low-grade glioma in the left SMA that caused a Parkinsonian syndrome, characterized by akinesia, rigidity, a resting tremor and micrographia (Straube and Sigel, 1988). This lesion extended more inferiorly in the corpus callosum, but it does illustrate a common denominator in the SMA syndrome and PD (Dick et al., 1986).

**Figure 3:**
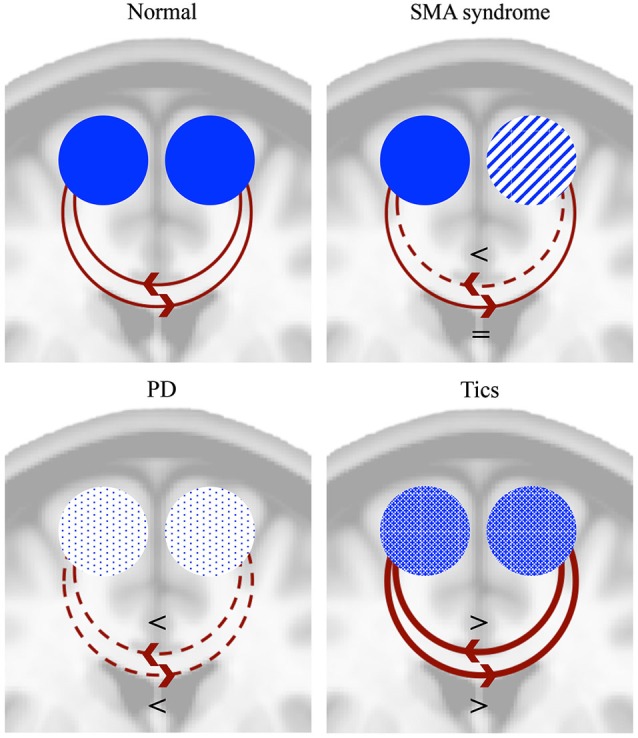
**Proposed mechanisms of modulation of the SMA in normal subjects, SMA syndrome, PD and tics**. The SMA can both positively and negatively modulate the contralateral SMA (Grefkes et al., 2008). In normal conditions this tonic interhemispheric balance may result in both initiation and inhibition of movements. In the SMA syndrome this balance is disturbed, leading to temporary lack of movements (akinesia) of the contralateral limbs and irreversible deficits of bimanual alternating movements. In PD, activity of both SMA’s is reduced, leading to akinesia and disturbances in bimanual alternating movements. Tics, however, result from bilaterally increased SMA activity. A disturbed interhemispheric balance may either aid in the suppression of tics or mediate the generation of tics. The functional schemes are projected on a coronal MNI brain section. = denotes unchanged modulation, < denotes decreased modulation, > denotes increased modulation.

Direct electrical stimulation of the SMA can lead to inhibition of movement or speech arrest, while it can also evoke movements, the urge to move or vocalizations (Penfield and Welch, 1951; Fried et al., 1991; Chauvel et al., 1996). Similarly, ictal speech arrest and vocalizations were seen in patients with SMA lesions (Ackermann et al., 1996; Wieshmann et al., 1997). From this perspective of opposite effects it is interesting to compare findings from the SMA syndrome with tics. Although the underlying pathophysiology is far from restricted to the SMA in patients with tics (Ganos et al., 2013), there are some interesting similarities with the SMA syndrome. Tics, as part of the Tourette syndrome, can be considered as movements that escape voluntary control (Jankovic, 1997). Typically they are preceded by a feeling of urge (Leckman et al., 1993) and can be voluntarily suppressed to some extent. Patients with Tourette syndrome show an increased resting state activity in the SMA compared to healthy subjects (Pourfar et al., 2011). There is a strong correlation in activation between the SMA and primary motor cortex during tics (Hampson et al., 2009), while activation of the SMA is positively correlated with tic severity (Wang et al., 2011; Ganos et al., 2014). Moreover, the SMA is active before tic onset (Bohlhalter et al., 2006). On the other hand, it is unclear whether the activity in the SMA is involved in tic generation or that it represents the effort of suppression of a tic. The SMA, together with a wider frontal network, is activated during the suppression of tics and is also more active during suppression of voluntary movements in patients with Tourette syndrome compared to healthy controls (Serrien et al., 2005). It thus seems from functional MRI studies (fMRI) that the normal system of inhibition, in which the SMA is involved, has adapted in order to suppress tics (Serrien et al., 2005). Inconsistent with this assumption, low-frequency (inhibitory) rTMS over the SMA leads to a reduction of tics (Chae et al., 2004; Mantovani et al., 2006, 2007; Kwon et al., 2011). Apart from tics, patients with Tourette syndrome frequently show echophenomena (Finis et al., 2012); automatic imitations that are presumed to be normal in the first year of life, but are considered as a complex tic when they reappear (Ganos et al., 2012). Interestingly, high-frequency rTMS of the SMA in healthy people can also induce echophenomena (Finis et al., 2013). An important remark concerns the idea that activation of the SMA as seen in fMRI studies can imply both positive and negative modulation, favoring the idea that the SMA has a causative role in the generation of tics instead of suppression of tics. While disturbed SMA activity in patients with the SMA syndrome and PD results in a lack of movements, changed/increased activity of the SMA in patients with tics is involved in the generation of movements. In the next paragraph an integrative explanation is proposed for this seemingly dualistic or “thermostatic” role of the SMA in initiation and inhibition upon direct electrical stimulation, in epilepsy and in tics and echophenomena. Figure 3 summarizes the proposed modulatory effects of both SMA’s in the SMA syndrome, PD and tics.

## Insight in SMA functioning

How should this apparent discrepancy between lack of movement initiation after lesions of the SMA and inhibition of movements due to an increased activity in the SMA be integrated? We recognize that any explanation remains hypothetical, but it may offer grip for further understanding. Lesions of the SMA are sometimes accompanied by temporary grasp reflexes or even an alien hand syndrome (Goldberg et al., 1981; Gelmers, 1983; McNabb et al., 1988; Rostomily et al., 1991; Zentner et al., 1996; Krainik et al., 2001), although this is accompanied by damage to the anterior cingulate cortex and anterior corpus callosum respectively (De Renzi and Barbieri, 1992; Doody and Jankovic, 1992; Feinberg et al., 1992; Scepkowski and Cronin-Golomb, 2003).

As previously mentioned, stimulation of the SMA can evoke movement initiation as well as an arrest in movements. Moreover, the SMA is active during the sight of a graspable object (Grèzes and Decety, 2002). While electrical stimulation of the primary motor cortex not only leads to muscle twitches but can evoke complex, coordinated movements of multiple joints (Graziano et al., 2002), the SMA seems to have a different role in more complex motor planning. Previously, a leading opinion was that activity in the SMA was related to volitional, internal generation of movements, but it has more recently been shown that the SMA has a function in both internally and externally generated movements (Tanji et al., 1985; Cunnington et al., 2002). Currently, activation in the pre-SMA has been related to volition (Nachev et al., 2005). Sumner et al. nicely demonstrated that the SMA is in fact implicated in automatic effector-specific inhibition of motor plans (Sumner et al., 2007; Boy et al., 2010). This is substantiated by the connections of the SMA with the subthalamic nucleus forming a hyperdirect pathway that suppresses thalamocortical circuits, which leads to a cessation of movement (Nambu et al., 1996). In the light of the akinetic deficits following resection of the SMA, but also in PD, this does not provide a full explanation. Possibly, the strong interconnection between the two SMA’s (Rouiller et al., 1994; Wiesendanger et al., 1996) enables the maintenance of a tonic interhemispheric balance involved in the initiation but also inhibition of movements. This balance can lead to both excitatory and inhibitory activity upon cortical and subcortical stimulation, with a preponderance for inhibition (Mikuni et al., 2006; Schucht et al., 2013). Regions that lead to cessation of movement after stimulation have been called negative motor areas (NMA; Lüders et al., 1995). There seems to be a remarkable lower incidence of the motor SMA syndrome and disturbance of bimanual function when leaving subcortical white matter NMAs originating from the SMA intact during resection of tumors in this area (Schucht et al., 2013; Rech et al., 2014). As seen from the localization of the stimulation sites it is probable that the NMA’s include both white matter tracts that connect the two opposite SMA’s as well as other tracts originating from the SMA. For example, transiently disturbed motor initiation has been correlated with a resection close to the fronto-striatal tract (also called subcallosal fasciculus) that connects the SMA with the caudate nucleus (Kinoshita et al., 2014), providing evidence that this is an important outflow tract of this network. Moreover, direct stimulation of this tract also induces initiation disorders (Duffau et al., 2002).

Furthermore, our hypothesis is consistent with the fact that the SMA can both initiate and suppress movement after a sensory instruction (Kurata and Tanji, 1985; Tanji and Kurata, 1985). The SMA is able to achieve this by both promoting and suppressing primary motor cortex activity (Grefkes et al., 2008), through activity prior to activation of the primary motor cortex (Vidal et al., 2003). This explanation seems also consistent with the role of the SMA and pre-SMA in linking conditional rules to actions (Nachev et al., 2008) and the role of the SMA in the temporal organization of movements (Shima and Tanji, 1998, 2000). Unilateral lesioning shifts this balance towards a lack of initiation, which can be restored once a new balance has been created. The fact that patients with the SMA syndrome can move upon strong encouragement is likely to be the result of compensatory circuits.

This tonic regulation can also explain the deficit in bilateral alternating movement patterns following unilateral lesioning of the SMA, while mirror movements are preserved (Bleasel et al., 1996). It has been shown that integrity of the parts of the corpus callosum that connect both SMA’s correlates with better asynchronous bimanual finger-thumb opposition (Johansen-Berg et al., 2007). Alternating movements require a difficult balance between inhibition of movement followed by initiation of movement, especially when this has to be done rapidly with two hands. Anti-phase movements require effective contralateral suppression, which is disturbed after resection of the SMA, but also in PD. Apparently, both SMA’s are necessary to perform alternating movements.

The tonic interhemispheric balance could also be an explanation for the above-mentioned apparent disparity between activation of the SMA that leads to suppression of tics, while inhibition of the SMA reduces tic frequency and activation of the SMA in healthy controls can lead to echophenomena.

Our model has a focus on the initiation and inhibition of movements with a special interest in bimanual alternating movements. It has been shown that there are more NMAs, for example in/near other premotor areas (Mikuni et al., 2006). It is unclear whether the outflow of these areas projects to the SMA or that this is a separate system. It would be interesting to see if the SMA’s are the final node in determining initiation or inhibition of movement. In this, alternating movements are apparently most demanding, requiring both SMA’s. Our model is restricted to the interaction between the SMA’s. Evidently, the SMA is part of a larger network, with rich connections to other cortical and subcortical areas.

## Conclusion

The SMA syndrome is an intriguing syndrome, characterized by temporary dysfunction, that helps to obtain useful insights in the function of the SMA and its embedment in neuronal circuits. The main aim of this article was not to write a comprehensive review on the function of the SMA, as these are available. Here we summarized the findings from previous studies regarding the SMA syndrome and showed that there are analogs with seemingly very different disorders such as PD and tics. Combining these findings, we propose that the SMA is involved in both the initiation and suppression of movements, maintaining a tonic interhemispheric balance. In this physiological context, the presentation of temporary deficits of the SMA syndrome supports the view that the healthy SMA can compensate for the functional impairment inflicted by the affected SMA. This concept is further supported by the persistent impairment of performing bimanual anti-phase movements, a motor condition in which such compensation apparently fails due to a strong simultaneous demand on both SMA’s.

## Author contributions

Conception of the work: A. R. E. Potgieser.

Design of the work: A. R. E. Potgieser, B. M. de Jong, M. Wagemakers, E. W. Hoving, R. J. M. Groen.

Interpretation of the work: A. R. E. Potgieser, B. M. de Jong, M. Wagemakers, E. W. Hoving, R. J. M. Groen.

Drafting the work: A. R. E. Potgieser.

Revising critically for important intellectual content: A. R. E. Potgieser, B. M. de Jong, M. Wagemakers, E. W. Hoving, R. J. M. Groen.

Final approval of the version to be published: A. R. E. Potgieser, B. M. de Jong, M. Wagemakers, E. W. Hoving, R. J. M. Groen.

Agreement to be accountable for all aspects of the work: A. R. E. Potgieser, B. M. de Jong, M. Wagemakers, E. W. Hoving, R. J. M. Groen.

## Conflict of interest statement

The authors declare that the research was conducted in the absence of any commercial or financial relationships that could be construed as a potential conflict of interest.

## Footnotes

^1^This is freely available on www.fmrib.ox.ac.uk/fsl
