# Erratum on: Insights from the supplementary motor area syndrome in balancing movement initiation and inhibition

**DOI:** 10.3389/fnhum.2015.00019

**Published:** 2015-01-22

**Authors:** Frontiers Production Office

**Affiliations:** Frontiers Production Office, FrontiersSwitzerland

**Keywords:** supplementary motor area (SMA), supplementary motor area syndrome, akinetic mutism, neurosurgery, Parkinson's disease, tic disorders

Reason for Erratum:

The first sentence of Figure 1 caption was applied to Figure 2 caption along with the footnote that was supposed to be in the caption for Figure 1, due to a typesetting error. This error does not change the scientific conclusions of the article in any way. The publisher apologizes for this error and the correct version of both Figures 1, 2 with their corrected captions appears below.

**Figure 1:**
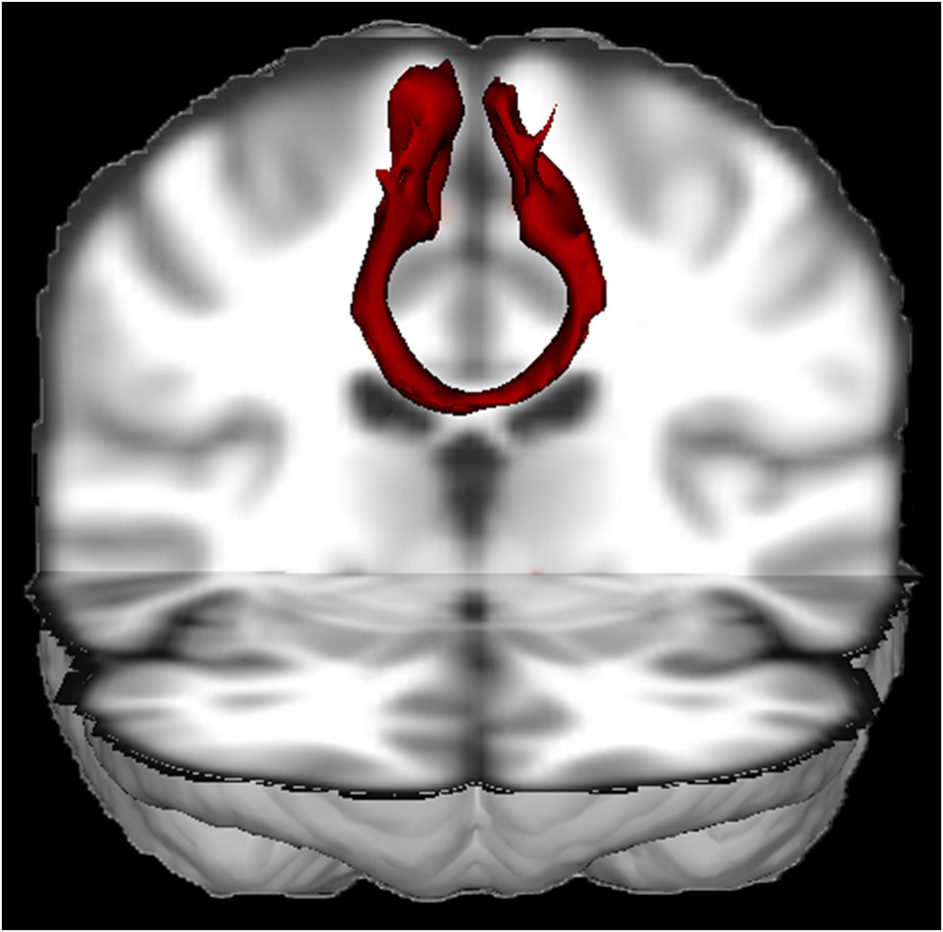
**3D view of the probabilistic tractography between both SMA's from a single healthy subject (made with FSL)^1^**. The tractography result was transformed to Montreal Neurological Institute (MNI) space. This figure nicely illustrates that the SMA's are densely interconnected through the corpus callosum.

**Figure 2:**
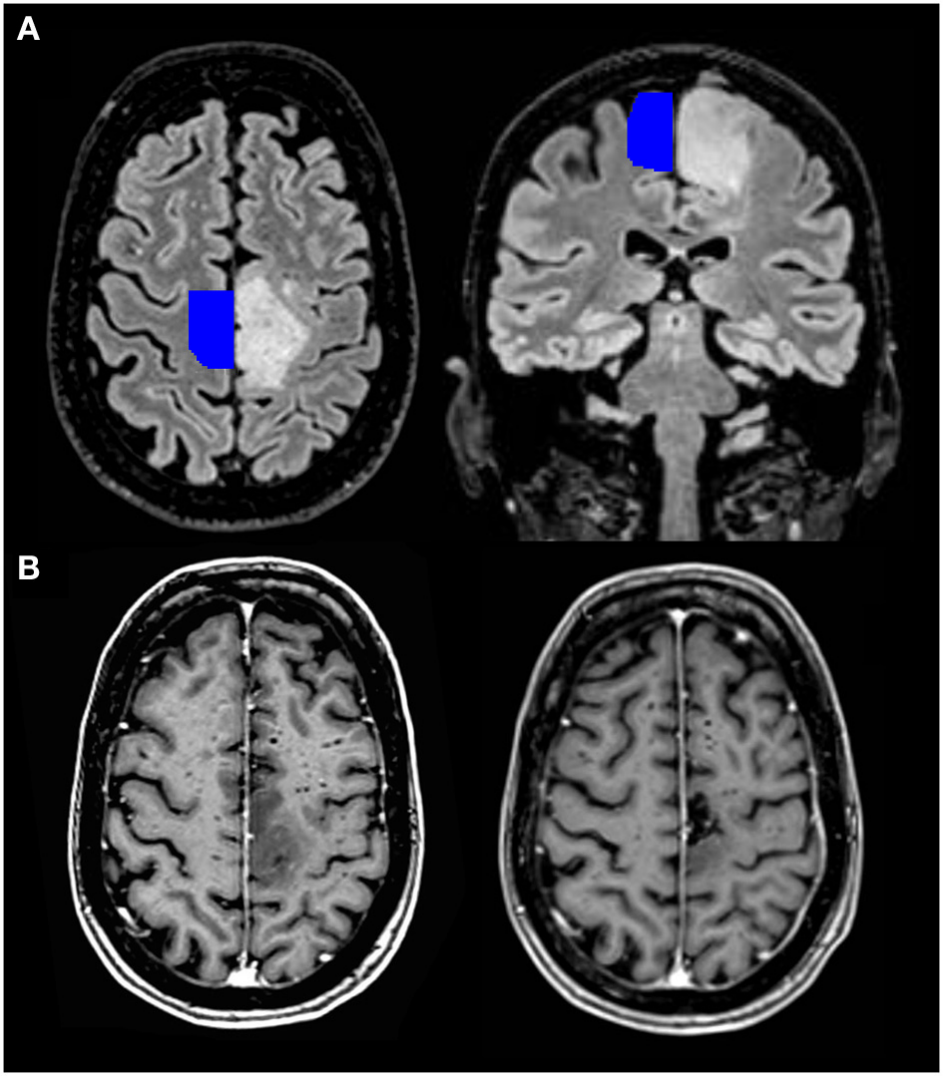
**Pre- and post-operative MRI scan of a 64-year-old patient with a diffuse astrocytoma (WHO grade II) in the left SMA. (A)** Transversal and coronal T2-weighted FLAIR images, with an SMA template projected on the healthy hemisphere. The latter is freely available and derived from a large meta-analysis describing the location of the sensorimotor areas (Mayka et al., 2006). **(B)** Transversal images after gadolinium contrast from the same patient before (left lower corner) and three months after the operation (right lower corner). She had a complete motor loss on the right side after the operation, which quickly recovered.

## Footnotes

^1^This is freely available on www.fmrib.ox.ac.uk/fsl.

